# ADAPTATION OF THE PPC APPROACH FOR HISPANIC OLDER ADULTS

**DOI:** 10.1093/geroni/igad104.1419

**Published:** 2023-12-21

**Authors:** Rafael Samper-Ternent, Gilberto Perez, Melissa deCardi Hladek, Carolina Fonseca

**Affiliations:** UTHealth Houston, Houston, Texas, United States; UTHealth Houston, Houston, Texas, United States; Johns Hopkins School of Nursing, Baltimore, Maryland, United States; Harvard Medical School, Boston, Massachusetts, United States

## Abstract

Older Hispanics have a high prevalence of MCC, contributing to a higher risk for dementia. As a result, there is a rapid increase in the number of young Hispanics assuming caregiver roles. The caregiver role is a cultural expectation among Hispanics and caregiving by the family is preferred over professional caregiving. There is limited information on how the unique context of Hispanics impacts the dyadic interactions between older adults and their care partners. There is a gap in the literature on how health values among Hispanics frame their healthcare goals and preferences and whether care partner involvement impacts the identification of these goals and preferences. There is also a limited number of evidence-based interventions that can help shift the current healthcare paradigm from clinical-guideline-based care to patient-centered care for underrepresented older adults. Thus, we used the Heuristic Framework to culturally adapt PPC for Hispanics with MCC and cognitive impairment living in Texas and embed it in two Geriatrics Clinics. We will describe the adaptation and implementation process and share findings from a pragmatic pilot study with 25 older Hispanics that participated in the PPC approach. We will share the outcomes from this pilot and the feedback received from patients, caregivers, and clinicians on the implementation of PPC at these clinics. We will conclude by sharing the current plans to expand the outreach of PPC for diverse older adult populations.

